# Refined Appearance Potential Mass Spectrometry for High Precision Radical Density Quantification in Plasma

**DOI:** 10.3390/s22176589

**Published:** 2022-08-31

**Authors:** Chulhee Cho, Sijun Kim, Youngseok Lee, Wonnyoung Jeong, Inho Seong, Jangjae Lee, Minsu Choi, Yebin You, Sangho Lee, Jinho Lee, Shinjae You

**Affiliations:** 1 Department of Physics, Chungnam National University, 99 Daehak-ro, Yuseong-gu, Daejeon 34134, Korea; 2 Samsung Electronics, Samsungjeonja-ro, Hwaseong-si 18448, Korea; 3 Korea Institute of Machinery & Materials, 156 Gajeongbuk-ro, Yuseong-gu, Daejeon 34103, Korea; 4 Institute of Quantum System (IQS), Chungnam National University, Daejeon 34134, Korea

**Keywords:** plasma, quadrupole mass spectrometer, radical density, quantification

## Abstract

As the analysis of complicated reaction chemistry in bulk plasma has become more important, especially in plasma processing, quantifying radical density is now in focus. For this work, appearance potential mass spectrometry (APMS) is widely used; however, the original APMS can produce large errors depending on the fitting process, as the fitting range is not exactly defined. In this research, to reduce errors resulting from the fitting process of the original method, a new APMS approach that eliminates the fitting process is suggested. Comparing the neutral densities in *He* plasma between the conventional method and the new method, along with the real neutral density obtained using the ideal gas equation, confirmed that the proposed quantification approach can provide more accurate results. This research will contribute to improving the precision of plasma diagnosis and help elucidate the plasma etching process.

## 1. Introduction

Low-temperature plasma is generated by an electric field in a space. Energetic electrons from the electric field hit neutrals, which divides them into reactive neutrals called radicals and charged particles [1,2]. These particles play individual roles in industry; charged particles physically affect a surface, while radicals chemically react with a surface. Such properties have led to the widespread use of plasma in semiconductor fabrication [3,4,5,6,7,8,9,10,11], agriculture [12,13,14], and medical treatment [15,16,17]. In particular, the role of plasma in semiconductor processing has gained importance in recent years along with the rapid growth of semiconductor performance. The current trend, therefore, is to seek high precision and refinement of the plasma process. However, due to the complexity of plasmas and inconsistencies in plasma analysis and processing from inaccurate plasma diagnosis, development remains based on trial and error. As the complexity of the plasma process increases, the trial-and-error approaches are reaching their limit, which has led to intense research to achieve more accurate plasma diagnostic methods.

The purpose of plasma diagnostics is typically to obtain particle densities and energy distributions. In particular, particle quantification has been extensively investigated [18,19,20,21,22,23,24]. As a representative example, the Langmuir probe is widely used to measure various plasma properties, such as electron energy distribution, electron density, plasma potential, and ion density [25]. Further, microwave probes, such as the cutoff probe, are used for electron quantification [26]. However, while electron quantification methods are diverse and accurate, the quantification of neutral species is difficult due to the low accuracy and high complexity of the current approaches. Since actinometry—a neutral quantification method used in optical emission spectrometry (OES)—requires a small amount of additional gas, it is difficult to use in the highly precise and refined semiconductor process [27]. Alternatively, the quadrupole mass spectrometer (QMS) is another tool that is widely used to obtain the density of plasma-neutral species [28,29,30]. A QMS measures the partial pressure of the neutrals without any extra gas in the chamber, providing an advantage over OES. Since the 1990s, steady efforts have been made by international standardization organizations to establish a standardized calibration procedure for QMSs, but as this is still in the first stage, quantification with a QMS remains difficult, especially in achieving high accuracy [31].

The main issue with neutral quantification via QMS is that additional neutral products are generated in the QMS. The QMS consists of three main components: an ionizer, a mass filter, and a detector. Electrons emitted by a filament in the ionizer are accelerated by the electric field between the positively biased anode grid and the negatively biased repeller; therefore, the electron energy is set to the voltage difference between the anode grid and the repeller. Neutrals are ionized by the accelerated electrons, with two cases of ionization by electron impact: direct ionization and dissociative ionization. Direct ionization means that molecules colliding with electrons lose their own electrons without decomposition. Direct ionization can be expressed as

$$A+e^{-}\to A^{+}+2e^{-},$$

where *A* is any neutral atom and $e^{-}$ is an electron. The direct ionization cross-section is zero below the threshold energy and rises linearly just above the electron energy. The maximum cross-section is usually reached at 50–100 eV [32]. Otherwise, dissociative ionization means ionization with the decomposition of molecules, which can be expressed as

$$AB+e^{-}\to A^{+}+B+2e^{-},$$

where *AB* is any molecule consisting of *A* and *B* atoms. As dissociative ionization can occur from the ions resulting from the direct ionization of other molecules, crucial errors can appear in the quantification of neutrals. Therefore, the separation of direct and dissociative ionization is one of the most important challenges to obtaining high precision neutral densities.

To separate the direct and dissociative ion signals, appearance potential mass spectrometry (APMS) is often used. APMS signals from a QMS, which sweeps the electron energy in the ionizer, are obtained by increasing the electron energy from below the ionization threshold energy to above the threshold energy and linearly fitting the signal to distinguish the direct and dissociative ionization signals. The energy dependence of the signals is eliminated by the linear fitting, allowing APMS to be used simply and briefly. However, the APMS signals curve up near the threshold energy, so no fitting standard has been confirmed. Therefore, the slope of the linear fitting values is changed with the fitting standard; consequently, neutral quantification with inaccurate fitting values can lead to enormous errors. The specifics of this method are described in Section 3.1 of this paper.

In this work, a refined APMS approach without a fitting process is suggested. This work compares the same neutral density in three different ways, as follows. First, the neutral density is calculated with the ideal gas equation for use as a reference. Second, the neutral density is obtained by the original APMS approach from the QMS signals gathered by sweeping the electron energies and conducting the fitting process. Third, the neutral density is acquired through the refined approach, which uses the same signals as the original APMS but with no fitting process.

The rest of this paper is organized as follows. Details of the experimental setup for APMS are described in Section 2. The specific methods and results for the original APMS method are given in Section 3.1, and the results of the refined APMS method are discussed in Section 3.2. Section 4 concludes the work.

## 2. Experimental Setup

**Figure 1:**
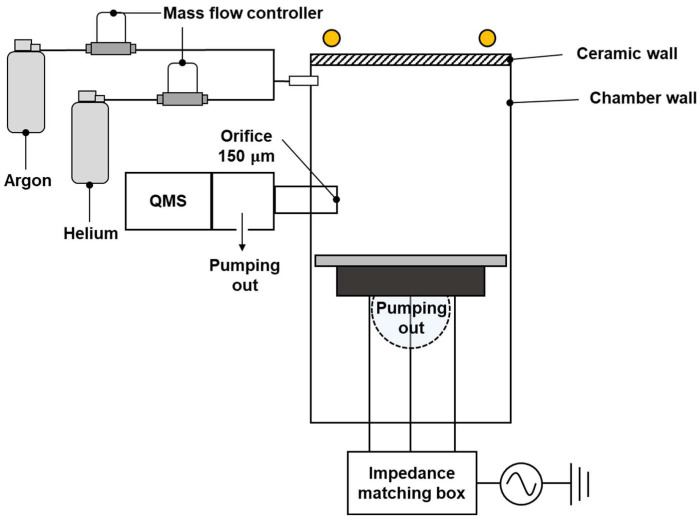
Experimental setup for *He* density measurement with a quadrupole mass spectrometer.

A schematic outline of the vacuum chamber is shown in Figure 1. Two mass flow controllers (LineTech Inc., Daejeon, Korea) injected 99.999% purity *Ar* and *He* gas at a maximum flow rate of 50 standard cubic centimeters per minute (sccm). The chamber was pumped out by a 99-L-per-min (L/min) rotary pump (Varian, Crawley, UK) that was connected to the center of the chamber body. The base pressure of the chamber was 5.3 Pascal (Pa), which was read by a Baratron gauge (MKS, Andover, MA, USA).

A QMS (Hiden Analytical, Warrington, UK, PSM) was installed in the chamber via a 150-μm orifice that separated the QMS chamber to create a high-level vacuum to remove neutral–neutral collisions in the QMS. The base pressure of the QMS chamber was below $1.3\times 10^{-5}$ Pa, which was read by a compact cold cathode vacuum gauge (Pfeiffer IKR 251, Pfeiffer Vacuum, Asslar, Germany). This high vacuum was sustained by a 300 L/s single turbo molecular pump (Edwards, Burgess Hill, UK) and a 200 L/min rotary pump (WSA, Gunpo, Korea).

When comparing the APMS approaches, a neutral density reference should first be obtained. In a vacuum chamber filled with inert gas, such as *Ar* and *He*, the density of the gas can be calculated from the pressure of the chamber using the ideal gas law because of the non-reaction of inert gas. The ideal gas law is expressed as [33]

(1) $$n=\frac{P}{kT}\;,$$

where n is the neutral density in the main chamber, P is the main chamber pressure, k is the Boltzmann constant, and T is the gas temperature. Here, the gas temperature was approximated to be room temperature (300 K). In this work, neutral densities for two cases were calculated. The conditions of the first case were 5 sccm *Ar* injection, 10.13 Pa main chamber pressure, and 2.45 $\times 10^{9}{\;m}^{-3}\;$*Ar* density. The conditions of the second case were 20 sccm *He* injection, 31.6 Pa chamber pressure, and 7.64 $\times 10^{9}{\;m}^{-3}$ *He* density. This *He* density was used for the theoretical value of the density. The QMS signals used in the APMS approaches were obtained under the same conditions as above. In other words, the QMS signals were obtained from two conditions, one with the chamber filled with 20 sccm *He* and the other with the chamber filled with 5 sccm *Ar*. Before the experiments, the QMS filament was heated for over 2 h, and the pressure of the chamber was kept at vacuum for more than 1 day. The QMS signals were obtained over 30 cycles for reproducibility, and the average values of the signals were used. To calibrate the QMS system, the built-in QMS calibration software RGAtune was used.

## 3. Results and Discussion

### 3.1. Original APMS Approach

**Figure 2:**
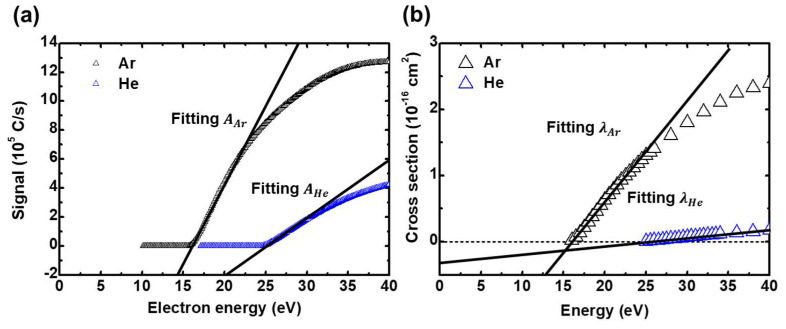
(**a**) *Ar* and *He* signals from the QMS. (**b**) *Ar* and *He* direct ionization cross-section.

The QMS displays signals by detecting the current generated by ions. Therefore, the expression of the QMS signal from the neutral species is given by [33]

(2) $$S=\beta t\left(\frac{m}{e}\right)\theta \left(\frac{m}{e}\right)l_{cage}I_{e}\sigma n_{ionizer},$$

where S is the QMS signal intensity, β is the extraction efficiency of the ions from the ionizer, t is the transmission efficiency of the quadrupole mass filter (which is a function of the ratio of mass to charge), θ is the detection coefficient of the channeltron detector, $l_{cage}$ is the ionizer length, $I_{e}$ is the electron current in the ionizer, σ is the cross-section of ionization, and $n_{ionizer}$ is the density of neutrals in the ionizer. As Equation (2) shows, neutrals can be detected in the QMS when becoming ions. Figure 2a shows the measured QMS signal of *Ar* at 10.13 Pa chamber pressure and *He* at 31.6 Pa chamber pressure, and Figure 2b shows the *Ar* and *He* direct ionization cross-section [32]. The signals are obtained by sweeping the electron energy of the ionizer from 10 eV to 40 eV. Referring to the cross-section, *Ar* becomes *Ar*+ after the electron energy exceeds 15.8 eV, and *He* becomes *He*+ after the electron energy exceeds 24.7 eV. Therefore, the signals appear after the electron energies increase above each threshold energy of neutral ionization. The signal curves up near the threshold energy, and after that, it linearly increases with electron energy. When the electron energy is higher than the threshold energy by 5–10 eV, the increase rate of the signal slightly declines. These signals are used for calculating the density via APMS.

The original APMS method is well known for measuring the radical species from plasma [33]. Singh et al. suggested the radical density quantification of X in plasma using Equation (2) as shown below [33]

(3) $$n_{X}^{on}=\left(\frac{A^{X\to X^{+}}}{A^{Ar\to Ar^{+}}}\right)\left(\frac{\lambda^{Ar\to Ar^{+}}}{\lambda^{X\to X^{+}}}\right)\left[\frac{t\left(m_{Ar^{+}}\right)\theta \left(m_{Ar^{+}}\right)}{t\left(m_{X^{+}}\right)\theta \left(m_{X^{+}}\right)}\right]n_{Ar}^{off}.$$

Here, as the purpose of this work was to compare *He* density, X is *He*, and A and λ were the linear fitting slope of the QMS signal and cross-section, as shown in Figure 2. The superscripts *on* and *off* of the density in Equation (3) indicate whether plasma is generated or not; note these superscripts in Equation (3) are excluded in this article since the QMS signal measurements were conducted not in a plasma environment but in a vacuum. We also note that Singh measured the oxygen atom density generated from plasma, while in the current work *He* density is unchanged with the plasma operating condition (*on* or *off*), so the plasma is not operated and $n_{X}^{on}$ is transformed to $n_{He}^{off}$. In addition, Singh stated that t(m)θ(m) for singly charged ions can be expressed as being proportional to $m^{-1}$, but also that it can change by instrument characteristics. Lee et al. recommended a calculation method for t(m)θ(m) using a standard gas, namely a gas mixture of various noble gas species [34]. Following this recommendation, f(m) as a function of t(m)θ(m), is given as

(4) $$f\left(m\right)=1.05\times 10^{7}e^{-0.028m}.$$

With this, Equation (3) can be rewritten as below,

(5) $$n_{He}=\left(\frac{A^{He\to He^{+}}}{A^{Ar\to Ar^{+}}}\right)\left(\frac{\lambda^{Ar\to Ar^{+}}}{\lambda^{He\to He^{+}}}\right)\left[\frac{f\left(m_{Ar}\right)}{f(m_{He)}}\right]n_{Ar}.$$

**Figure 3:**
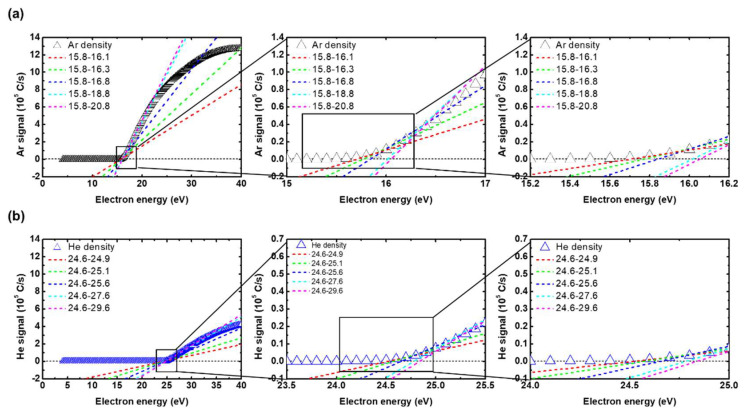
Different fitting results with different fitting ranges of the QMS signals for (**a**) *Ar* and (**b**) *He*.

Here, A and λ, which are suggested by Singh, should be calculated near the threshold energy. However, since QMS signals are not linear right above the threshold, the linear fitting results can differ significantly according to their fitting ranges. Figure 3a,b show different fitting lines with different fitting ranges of *Ar* and *He*, respectively. Each fitting range was determined to be from the threshold energy to some energy higher than the threshold by an arbitrarily chosen amount. The fitting ranges and resulting slopes obtained from the *Ar* and *He* signals are listed in Table 1. Ranges 1–5 correspond to fitting ranges of 0.3, 0.5, 1, 3, and 5, respectively. In Figure 3, it can readily be seen that the different fitting ranges cause various slopes of the fitting lines, leading to inaccurate density calculations. In addition, it should be noted that decisions on which range is the most suitable can also be altered by the axis scales of the plots, as shown in the insets of Figure 3a,b. This may possibly generate considerable errors in density quantifications from QMS signals as well. Table 2 lists the fitting slopes of the cross-section, obtained from only one range since the cross-sections linearly increase near the threshold energy.

**Figure 4:**
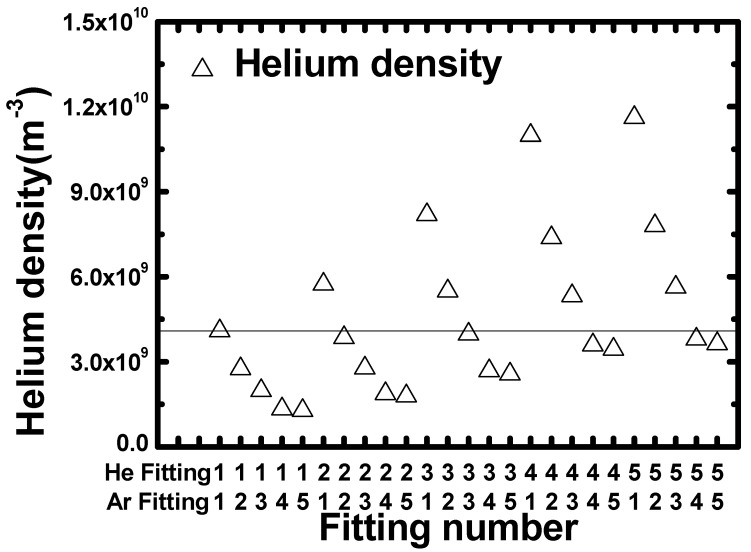
Plot of *He* densities calculated with each *He* and *Ar* fitting range. The horizontal line is the average value of the density.

By substituting the fitting results in Table 1 and Table 2 for *A* and λ*,* respectively, in Equation (5), and the *Ar* density obtained by the ideal gas law in Section 2 for $n_{Ar}$ in Equation (5), the *He* densities resulting from the different fitting ranges can be calculated. Figure 4 plots the calculated *He* densities from Equation (5). Note that $A^{Ar\to Ar^{+}}$ and $A^{He\to He^{+}}$ in Equation (5) each had five different values, as listed in Table 1 and Table 2, which resulted in a matrix multiplication of 25 total densities. As shown in Figure 4, *He* density increased as the fitting range for *He* was increased, while for *Ar* density decreased with an increase in fitting range. The average of the calculated *He* densities was $4.53\times 10^{9}\;m^{-3}$*,* which is similar to the theoretical *He* density calculated from the ideal gas law. The *He* densities calculated from the same *Ar* and *He* ranges (i.e., 1-1, 2-2, 3-3, etc.) were closer to the average value. This phenomenon occurs because the rates of change of $A^{He\to He^{+}}$ and $A^{Ar\to Ar^{+}}$ are similar.

### 3.2. Refined APMS

The *He* densities from the original APMS approach have a huge deviation, leading to insufficient precision. Because of the curvature of the signal near the threshold energy, it is hard to choose the correct fitting range, and this can cause errors. Therefore, a quantification method without the fitting process is suggested. Singh et al. [33]. stated that the QMS signal and direct ionization cross-section can be expressed from the linear fitting slope with energy dependence as below,

(6) $$S\left(E\right)=A\left(E-E_{i}^{X\to X^{+}}\right),$$

(7) $$\sigma \left(E\right)=\lambda \left(E-E_{i}^{X\to X^{+}}\right),\;$$

where *E* is the electron energy and $E_{i}$ is the direct ionization threshold energy. Therefore, to remove the fitting process, the refined quantification method neglects the slope through the fitting and directly uses the signal and cross-section with energy dependence. According to Equations (6) and (7), A and λ in Equation (5) can be substituted with S and σ. The refined equation is then expressed as below,

(8) $$n_{He}\left(E\right)=\left(\frac{S^{He\to He^{+}}\left(E\right)}{S^{Ar\to Ar^{+}}\left(E\right)}\right)\left(\frac{\sigma^{Ar\to Ar^{+}}\left(E\right)}{\sigma^{He\to He^{+}}\left(E\right)}\right)\left[\frac{f\left(m_{Ar}\right)}{f(m_{He)}}\right]n_{Ar}.$$

Here, S is the QMS signal and σ is the direct ionization cross-section. The measured QMS signals are 0.1 eV apart, and both *Ar* and *He* signals are calculated at the same energy. The cross-section is calculated by changing the interval of 1 eV in the low energy region to an interval of 0.1 eV through data preprocessing. Through the above formula, it is possible to calculate the density of *He* according to electron energy. Since the density of *He* is the value in the main chamber, there should be no change with electron energy sweeping.

**Figure 5:**
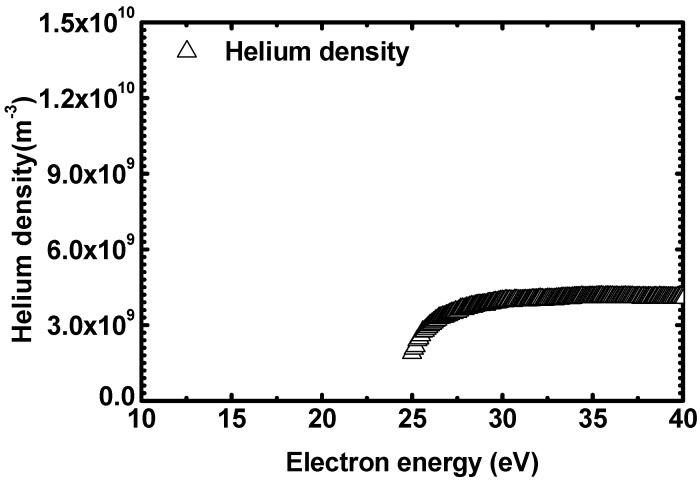
Plot of *He* densities obtained through the refined APMS method.

Figure 5 plots the *He* densities calculated with the refined APMS approach. As for the calculated densities, the signal appears beyond the electron energy of 24.7 eV (the threshold energy of *He* direct ionization) and it can be confirmed that it increases slightly as the energy increases, eventually saturating at around 27 eV. The initial value, which is the smallest one, appears to be approximately half of the saturated value. The measured *He* density can be obtained by averaging the densities in the region with the smallest change. Here, even if the result of the initial value with the largest error is included, there is no significant change in the average value because the region between the lowest point and the saturation point of *He* density is local compared to the saturation region. Therefore, *He* density from the refined APMS approach is obtained by averaging the entire electron energy area.

**Figure 6:**
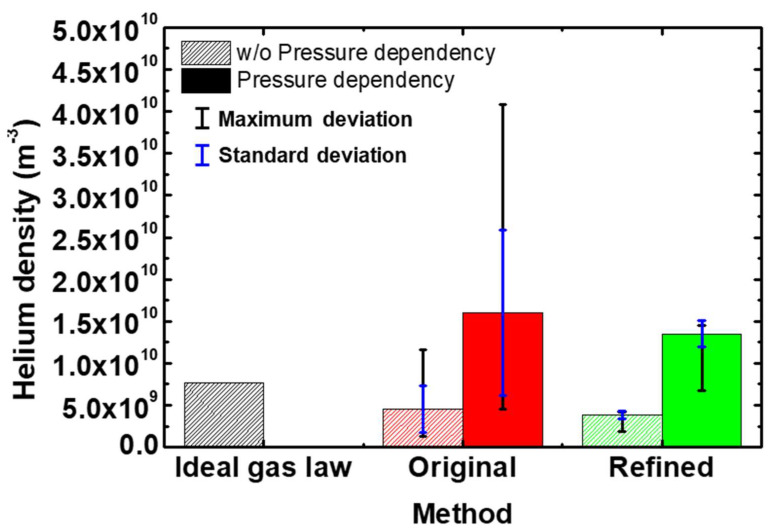
*He* densities from each quantification method. The maximum deviation is used.

**Figure 7:**
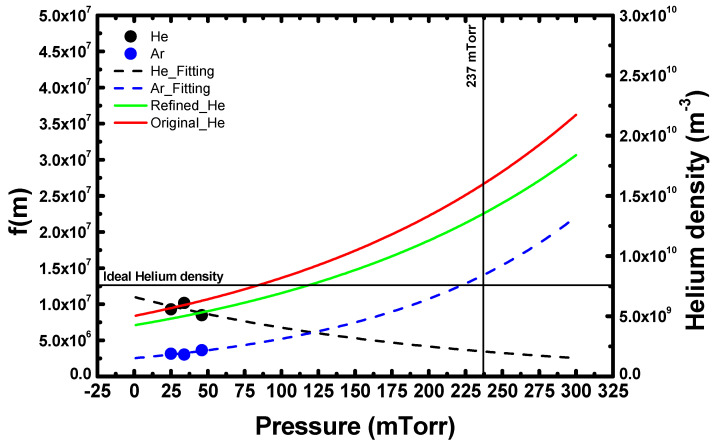
*He* density and f(m) with varying pressure. Black and blue circles are f(m), black and blue dashed lines are the fitted f(m), and green and red lines are the *He* density from the refined and original APMS methods, respectively.

In Figure 6, the *He* densities from each quantification method are shown. To check the maximum error, the maximum deviation was used. Therefore, the lower limits of the error bars were the minimum values in Figure 4 and Figure 5, while the upper limits were the maximum values of *He* density in Figure 4 and Figure 5. The density from the refined APMS method was $3.83\times 10^{9}{\;m}^{-3}$, a value that was further from the theoretical *He* density from the ideal gas law ($7.64\times 10^{9}{\;m}^{-3}$) compared to the value from the original APMS method ($4.53\times 10^{9}{\;m}^{-3}$). However, the original APMS results have both a larger maximum ($3.25\times 10^{9}{\;m}^{-3}$) and standard ($2.79\times 10^{9}{\;m}^{-3}$) deviation of *He* density due to fitting error compared to the refined APMS results with a smaller maximum ($1.93\times 10^{9}{\;m}^{-3}$) and standard ($4.47\times 10^{8}{\;m}^{-3}$) deviation. This demonstrates that the refined APMS approach provides more precise quantification results from QMS measurements than the original approach. Moreover, the accuracy of the refined APMS method can be further improved by considering the pressure dependence of f(m). The black and blue circles in Figure 7 are the f(m) values used at different pressures, where $f\left(m_{Ar}\right)$ and $f\left(m_{He}\right)$ were taken as averages. These values seem to be pressure-dependent, so the dashed lines in Figure 7 were obtained by fitting the circle values. Using the dashed lines, we obtained $f\left(m_{Ar},\;76\;\mathrm{mTorr}\right)$ and $f\left(m_{He},\;237\;\mathrm{mTorr}\right)$. Then the *He* density, which was re-calculated with $f\left(m_{Ar},\;76\;\mathrm{mTorr}\right)$ and $f\left(m_{He},\;237\;\mathrm{mTorr}\right)$, increased compared to before; the refined result, (1$.35\times 10^{10}{\;m}^{-3}$), was closer to the ideal result than that from the original approach (1.59$\times 10^{10}{\;m}^{-3}$). This is illustrated in Figure 6 with the solid plots. Therefore, the possibility of achieving higher accuracy with the refined APMS method exists.

## 4. Conclusions

In this paper, we proposed a refined APMS approach for quantifying radical densities in plasma. With a simple modification of the original APMS approach, the fitting process was eliminated, and the *He* density was obtained over the entire electron energy ranges. After averaging the *He* density of each electron energy, the *He* density was compared to the density from the ideal gas law. As a result, while the *He* density from the refined APMS method, $3.83\times 10^{9}{\;m}^{-3}$, was further from the density of the ideal gas law, (7.64 $\times 10^{9}{\;m}^{-3}$), than that from the original approach, ($4.53\times 10^{9}{\;m}^{-3})$, the deviation of the density from the refined method was much smaller than that from the original APMS method. Based on this, the refined APMS approach is believed to be applicable to neutral density quantification with higher precision. This method has some limitations. To obtain correct signals from the QMS, the QMS chamber pressure should be below $1.3\times 10^{-5}$ Pa, and highly reactive radicals, such as fluorine and chlorine, should be avoided to prevent chemical reactions with the filament. Additionally, to use either APMS method, a cross-section is required. If an algorithm that can be applied to existing equipment is developed in future research, such as APMS, it is expected to enable more precise and accurate measurement, which could lead to adoption in various fields that require precise plasma diagnosis, such as the semiconductor etching process.

## Figures and Tables

**Table 1:** Linear fitting slope of *Ar* and *He* signals from the QMS with different ranges.

Range	$A_{Ar}$	$A_{He}$
Range 1	35,420	12,427
Range 2	52,751	17,423
Range 3	73,067	24,875
Range 4	108,389	33,386
Range 5	113,050	35,307

**Table 2:** Linear fitting slope of the *Ar* and *He* direct ionization cross-section.

Range	$\sigma_{Ar}$	$\sigma_{He}$
Range 1	0.16181	0.01249

## Data Availability

The data presented in this study are available upon request from the corresponding author.
